# Reduced reactive hyperemia may explain impaired flow‐mediated dilation after on‐pump cardiac surgery

**DOI:** 10.14814/phy2.13274

**Published:** 2017-05-29

**Authors:** Hans H. Dedichen, Jonny Hisdal, Eirik Skogvoll, Petter Aadahl, Idar Kirkeby‐Garstad

**Affiliations:** ^1^ Department of Circulation and Medical Imaging Faculty of Medicine Norwegian University of Science and Technology Trondheim Norway; ^2^ Clinic of Cardiothoracic Surgery St Olav's Hospital Trondheim University Hospital Trondheim Norway; ^3^ CircuT (Circulation Research Group Trondheim) Trondheim Norway; ^4^ Section of Vascular Investigations Oslo University Hospital Oslo Norway; ^5^ Clinic of Anaesthesia and Intensive Care St Olav's Hospital Trondheim University Hospital Trondheim Norway

**Keywords:** Cardiac surgery, cardiopulmonary bypass, endothelial function, flow mediated dilation

## Abstract

In previous studies, Flow Mediated Dilation (FMD) was used to study the effect of cardiac surgery on endothelial function. This study investigated the effect of on‐pump cardiac surgery on FMD and reactive hyperemia. The FMD‐response and reactive hyperemia were measured in 25 patients the morning before‐ and the first morning after cardiac surgery. Brachial artery diameter and blood flow were measured with ultrasound at baseline before 5 min occlusion of the blood flow to the forearm, and continuously for 3 min after release of the occlusion. An exponential wash‐out model was fitted to the blood flow over time. Nineteen patients remained for final data analysis. Data are mean ± SEM. The FMD response was reduced after surgery from 3.3 ± 0.5% to 1.4 ± 0.6% (*P* = 0.02). Max blood flow after cuff release was reduced from 342 ± 30 mL preoperatively to 305 ± 30 mL postoperatively (*P* < 0.00) and fell toward baseline significantly quicker; preoperative half‐life was 36 ± 2.4 sec. versus 29 ± 1.9 sec postoperatively (*P* < 0.00). Resting blood flow was reduced from 84 ± 9 mL/min to 66 ± 9 mL/min, (*P* < 0.00). Brachial artery baseline diameter was unaffected by coronary artery bypass surgery (*P* = 0.3). The observed reduction in brachial artery FMD after surgery, by previous authors taken to represent endothelial dysfunction, may at least partly be due to reduced hyperemic flow postoperatively. In studies where FMD is measured on multiple occasions, flow data should also be included. Reduced postoperative blood flow to the arm may indicate regional differences in vascular resistance after cardiac surgery.

## Introduction

The vascular endothelium participates in the regulation of vascular tone and tissue perfusion. Endothelial dysfunction is associated with increased cardiovascular risk and with increased risk of complications after major surgery (Schier et al. 2012, 2014; Matsuzawa et al. 2015). It is suspected that surgical trauma and “stress” may cause a transient deterioration in endothelial function, which in turn may contribute to postoperative vascular dysfunction (Celermajer et al. 1992; Ruel et al. 2004; Hu et al. 2013; Sondergaard et al. 2015).

Several methods are used to assess vascular endothelial function. Flow‐mediated dilatation (FMD) is currently considered to be the gold standard. FMD is noninvasive, safe, and feasible for clinical use. FMD uses ultrasound imaging to measure the dilation of the brachial artery during reactive hyperemia. Reactive hyperemia occurs when a five minutes occlusion of forearm blood flow is released and is due to vasodilatation caused by local metabolites affecting arterioles in the occluded forearm.

Two previous studies used FMD to assess the very early postoperative effect of on‐pump cardiac surgery on endothelial function. The results in coronary artery bypass grafting (CABG) patients were contradictory. Morelos et al. (2001) concluded that no further impairment occurred postoperatively. Chello et al. (2005) concluded that endothelial function was impaired after on‐pump surgery and that statin treatment resulted in a relative protection.

The FMD method has developed since Morelos and Chello used it more than 10 years ago. Newer ultrasound scanners can sample 2D images and Doppler shift signals simultaneously for several minutes, and it is now recommended to include Doppler flow measurements in the FMD test to assess the magnitude of reactive hyperemia (Thijssen et al. 2011). This questions the validity of previous results regarding the impact of on‐pump cardiac surgery on endothelial function. We, therefore, used the current methodology to compare preoperative and postoperative FMD in a prospective study. We hypothesized that FMD was maintained after on‐pump cardiac surgery.

## Methods

### Patients

Twenty‐five patients, who were admitted for elective coronary artery bypass surgery at St. Olav's Hospital, Trondheim, Norway from December 2013 to April 2014, were included in this study. The inclusion criteria were planned coronary artery bypass surgery with the use of extracorporeal circulation and oral and written consent to participate in the study. Exclusion criteria were unstable coronary artery disease, severe impairment of left ventricular function, significant valve disease, heart failure, renal disease, diabetes mellitus, connective tissue disease, nitrate treatment, renin–angiotensin system inhibiting drugs, calcium channel blockers, unstable postoperative circulation, ongoing postoperative bleeding, and severe postoperative nausea or vomiting. Patient characteristics are presented in Table 1.

**Table 1:** Patient characteristics

Variable	
Age (year)	68 (8)
Male sex [%]	17 [89]
Height (cm)	177 (7)
Weight (kg)	86 (15)
EF echo (%)	53 (7)
Current smokers [%]	5 [26]
NYHA class 2 [%]	5 [26]
NYHA class 3 [%]	14 [74]
Tree vessel disease [%]	16 [84]
Left main stem stenosis [%]	3 [17]
Hypertension [%]	5 [26]
Beta blocker [%]	14 [74]
Statin [%]	19 [100]

NYHA, New York Heart Association. Values are mean (SD) and number [%].

The patients received written and oral information about the study before consenting to participate. The study was approved by the Regional Ethics Committee for Mid‐Norway (Medisinsk teknisk forskningssenter, 7489 Trondheim, Norway, REK Midt 2013/1758) on 25. October 2013 and was registered at ClinicalTrials.gov (NCT02001090).

### Patient treatment

The patients received treatments that are standard for our institution. General anesthesia was induced with diazepam, thiopental, fentanyl, and cisatracurium and was maintained with isoflurane and fentanyl. During cardiopulmonary bypass (CPB) patients were sedated with propofol. The CPB circuit consisted of a roller pump and a microporous polypropylene hollow fiber oxygenator (Medtronic Affinity NT, (Medtronic, Minneapolis, Minnesota)) with an open reservoir (Medtronic Affinity CRV). The oxygenator and tubing were heparin coated, and the circuit was primed with 1700 mL Ringer's acetate containing 7500 U of heparin. Nonpulsatile flow at a flow rate of 2.4 L/min/m^2^, alpha‐stat blood gas control and cooling to 34°C in venous blood was used. During CPB, activated clotting time (ACT) was maintained greater than 480 sec by an initial administration of heparin (Leo, Copenhagen, Denmark) 300 U/kg, and additional doses were administered as indicated by the actual ACT. Cardiotomy suction was used when ACT was longer than 480 sec. After CPB, heparinization was reversed with protamine sulfate at a 1:1 ratio. The heart was arrested by cross‐clamping the ascending aorta and infusion of cold St Thomas’ Hospital cardioplegia solution. Standard revascularization included anastomosing the left internal thoracic artery to the left anterior descending artery and placing the vein grafts from the ascending aorta to the circumflex artery and the right coronary artery.

### Study protocol and measurements

**Figure 1:**
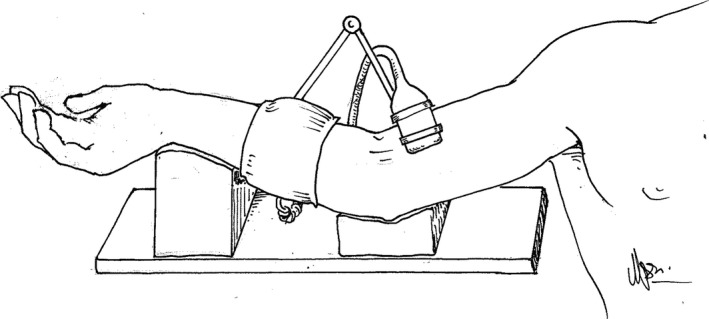
Illustration of the mechanical arm for the transducer and the support for the arm of the patient.

The preoperative test was made in the morning on the day of surgery. Premedication was not given before the test was finished. The postoperative test was performed in the intensive care unit the first morning after the operation. The patients were fasting and subject to bed rest. Drug infusions were stopped 30 min or earlier before the postoperative test. The test procedure was identical in the preoperative and postoperative tests. Patients were lying in bed with their upper body slightly elevated and the right arm firmly supported by gel pads on a specially made table. The ultrasound transducer was positioned over the brachial artery three to five cm proximal to the elbow crest. The transducer position was marked with a pen to facilitate similar positioning in the postoperative test. A mechanical arm attached to the table held the transducer over the brachial artery, and the position was fine‐tuned during measurements with a micrometer screw (Fig. 1). A pneumatic cuff (Hokanson SC5, Hokanson, Bellevue, Washington, USA) was placed on the lower arm and was inflated to a minimum pressure of 250 mmHg for five minutes. 30 sec baseline measurements were made before inflation of the cuff. 2D images and Doppler signals were recorded continuously for three minutes after cuff deflation to assure recording of reactive hyperemia and dilation maximum and return to normal. Arterial blood pressure was measured with a blood pressure tourniquet and stethoscope after the completion of the FMD measurements. The ultrasound measurements were made using a 12 MHz linear transducer connected to a Vivid 7 ultrasound machine (Vingmed GE, Horten, Norway). The Doppler beam‐vessel insonation angle was adjusted to be <60° to ensure valid shear rate calculations as suggested in Thijssen et al. (2011). The sample volume was adjusted to measure across the lumen, and the mean velocity across the cross‐section of the lumen was calculated. Two‐dimensional images and the blood flow velocity were recorded before cuff inflation and for three minutes after cuff deflation. Before automatic offline analysis of the brachial diameter and flow, the 2D images were down‐sampled to one picture for each heartbeat that was triggered by the ECG R‐wave. Software developed in‐house down‐sampled the 2D images. Automatic offline analysis of flow, brachial artery diameter, and FMD was performed using the Brachial Analyzer for Research (Medical Imaging Applications, LLC, Coralville, Iowa, USA).

### Data analysis and statistics

Data are expressed as the mean with standard deviation (SD) for descriptive purposes, or mean with standard error of the mean (SEM) when group differences are of interest. FMD pre‐and postoperatively were compared using paired samples’ t‐test. An exponential wash‐out model with three parameters was fitted to the brachial artery flow data: max hyperemic flow (starting value), resting flow (horizontal asymptote as time →∞), and half‐life; estimated under pre‐and post‐operative conditions. Assuming that cuff inflation/deflation does not have lasting effects, pre‐cuff inflation flow values were shifted along the time line by +6 min after cuff deflation to aid in determining the resting flow asymptote. To accommodate heteroscedasticity (i.e., increasing variance with increasing value), a variance function was included to achieve near normality of the residuals; observations were visually inspected and the model found to fit well. Estimation of parameters and random effects for max flow and resting flow in each individual was done using the package *nlme* (nonlinear mixed effects) of the software R version 3.3.0 (R Core Team, 2016). *P* < 0.05 was taken to indicate statistical significance. The number of patients required for the study was estimated from the studies by Morelos and Chello. Chello et al. reported a preoperative FMD of 4.7 (SD = 1.8) and a postoperative FMD of 2.8 (SD= 1) (statin group). In the data from Morelos et al., there was a correlation between the preoperative and postoperative FMD of −0.013. Using a paired a paired samples t‐test, 12 patients were necessary to demonstrate a difference in FMD response of 2% with a significance level of 0.05 and a power of 80%. To allow for potential dropouts and evaluation of blood flow influence on FMD we included 25 patients.

## Results

Twenty‐five patients were included in the study (Table 1). All patients had severe coronary artery disease verified by angiogram and underwent coronary artery bypass grafting with CPB (Table 2). There were no complications during or after testing, but one patient had a bruise under the cuff. None of the patients found the forearm occlusion painful. Six of the 25 patients were excluded from the final analysis because of unsatisfactory quality of the preoperative or postoperative ultrasound images (Hu et al. 2013) or because of noradrenaline infusion at the time of the postoperative test (Atkinson et al. 1985). Nineteen patients remained for the final data analysis. Fourteen of the nineteen patients were preoperatively on *β*‐blockers, and all patients were treated with statins. In the ICU, they were treated with cardio‐vasoactive drugs at the discretion of the attending physician. From admission to the ICU to the next morning 17 patients at some point received noradrenaline infusion and two patients received natrium nitroprusside infusion, both of which have a half‐life shorter than three minutes. Tree patients were given labetalol infusion; it has a half‐life of four hours. In one patient the labetalol infusion was stopped more than 12 h prior to the postoperative test, and in the other two, it was continued until the postoperative morning. All cardio‐vasoactive infusions were stopped at least 30 min before the start of the postoperative test and no cardio‐vasoactive drugs were given in this period.

**Table 2:** Patient treatment

Variable	
CPB time (minutes)	80 (26)
XC‐time (minutes)	53 (19)
Lowest rectal temperature on CPB (°C)	34.8 (0.7)
Number of vascular grafts	3.6 (1.2)
No of patients who received blood transfusion [%]	4 [21]
Postoperative fluid balance (mL)	3329 (1687)

CPB, cardiopulmonary bypass; XC‐time, myocardial ischemia time; Values are mean (SD) and number [%].

**Figure 2:**
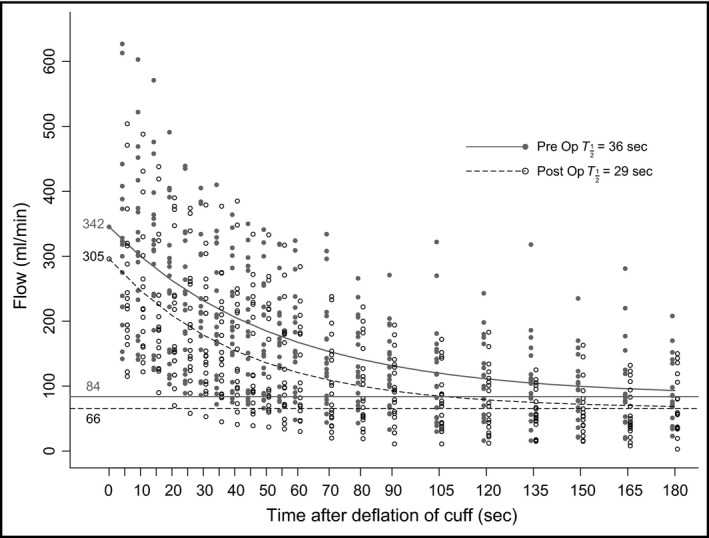
Postoperatively reduced brachial artery blood flow**.** Ultrasound Doppler measurements showed reduced blood flow at rest and during post‐ischemic reactive hyperemia (*P* = 0.000 and *P* = 0.003, respectively). The half live of reactive hyperemia was significantly shorter after surgery (*P* = 0.009).Dots are preoperative and circles are postoperative. Continuous line is estimated preoperative blood flow, and dashed line is estimated postoperative blood flow.

Flow‐mediated dilatation of the brachial artery was reduced from 3.3 ± 0.5% to 1.4 ± 0.6% (*P* = 0.02), after surgery (Table 3). A significant reduction in the maximum brachial artery blood flow immediately after cuff deflation after cuff occlusion (shear stimulus) was also observed, from 342 ± 30 mL to 305 ± 30 mL (*P* = 0.003) (Fig. 2). Furthermore, the brachial artery resting blood flow was also reduced after surgery; from 84 ± 9 mL preoperatively to 66 mL ± 9 after surgery (*P* = 0.00) (Fig. 2). The ratio between max and rest flow was not influenced (4.1 pre‐ vs. 4.6 postoperatively). The brachial artery flow also returned more quickly toward resting values after surgery (reactive hyperemia half‐life) 36 ± 2.4 sec pre‐ versus 29 ± 1.9 sec postoperatively (*P* = 0.009). The brachial artery baseline diameter was not affected by the coronary bypass surgery. The mean diameter of the brachial artery measured at rest before cuff inflation was 4.15 mm (0.17) before and 4.28 mm (0.15) after surgery (*P* = 0.3). The systolic and diastolic blood pressures were significantly reduced in the postoperative tests (*P* = 0.04 and 0.001, respectively).

**Table 3:** Results

Variable	Preop	Postop	*P*‐value
Brachial artery baseline diameter (mm)	4.15 (0.17)	4.28 (0.15)	0.3
Brachial artery baseline blood flow (mL/min)	84 (9.2)	66 (8.9)	0.00
Maximal reactive hyperemia (mL/min)	342 (30)	305 (30)	0.00
T ½ reactive hyperemia (s)	36 (2.4)	29 (1.9)	0.00
FMD (%)	3.3 (0.5)	1.4 (0.6)	0.02
Systolic blood pressure (mmHg)	127 (3.3)	114 (3.2)	0.04
Diastolic blood pressure (mmHg)	72 (2.6)	56 (1.8)	0.001
Mean arterial pressure (mmHg)	90 (2.4)	75 (1.9)	0.001
Hematocrit (%)	45.6 (0.9)	31.1 (0.6)	0.001

FMD (Flow Mediated Dilation). Values are mean (SEM).

## Discussion

The most important finding in this study is that both resting blood flow and reactive hyperemia were considerably reduced after the operation. The postoperative reduction in peak hyperemic blood flow and the significantly shorter half‐life of hyperemic flow mean that shear force on the vessel wall was reduced in the postoperative test. Furthermore, hematocrit, the single most important determinant of blood viscosity was reduced, contributing to a further reduction in shear forces (Giannattasio et al. 2002). Also, resting brachial artery diameter was unchanged from pre‐ to postoperative test. These findings corroborate that a significant postoperative reduction in vessel wall shear forces occurred. The reductions in shear forces may at least in part explain the observed reduction in FMD from 3.4% to 1.4% and postoperative reduction in FMD does not support the theory of endothelial function being impaired during surgery with CPB. The low preoperative FMD indicate that our patients, who suffer from severe vascular disease, had endothelial dysfunction. Regarding postoperative FMD our results are in good accordance with those of Chello et al. (2005) who demonstrated postoperatively reduced FMD in cardiac surgery patients. However, the present study demonstrates that the postoperative reduction in FMD does not necessarily indicate a postoperative deterioration in endothelial function.

Since the introduction of the FMD method in 1992 by Celermajer et al. (1992) simultaneous measurement of FMD and forearm reactive hyperemia has been introduced as an alternative indicator of cardiovascular risk. Several attempts have been made to calculate how much FMD could be expected to result from a given reactive hyperemia. However, a mathematical correction for the differences in reactive hyperemia is not straightforward because blood velocity, artery diameter, and blood viscosity must be included and laminar blood flow along the vessel wall must be assumed. The guidelines for FMD measurement published by American Journal of Physiology state that the use of ratio normalization (FMD/shear) is presently unresolved and the guidelines conclude that, at this time, it is not possible to recommend a method for correcting for the differences in shear (Atkinson et al. 1985). We, therefore, abstain from the use of FMD normalized for shear which is reported by some other investigators (Sangalli et al. 2015).

Furthermore, our finding of postoperatively reduced brachial artery blood flow provides insight to the circulation of cardiac surgery patients. Resting blood flow and reactive hyperemia were reduced by approximately 20% and 10%, respectively after the operation. At the same time mean arterial blood pressure was reduced by approximately 17%. This indicates that the vascular resistance in the arm is maintained or slightly increased from before to after the operation. Previous studies published by our research group have shown that similar patient groups had increased cardiac output (CO) and reduced systemic vascular resistance on the first morning after cardiac surgery and it is reasonable to assume that CO was postoperatively increased in this study as well (Kirkeby‐Garstad et al. 2005, 2006). However, as CO and central venous pressure were not measured in the present study we are not able to provide data on regional peripheral vascular resistance in the arm or on systemic vascular resistance.

In a review of vasomotor function after cardiac surgery Ruel et al. (2004) claim that peripheral vascular resistance in the skeletal muscle bed is reduced after cardiac surgery. A possible explanation for the discrepancy between our findings and the view held by Ruel et al. may be that our study was made 15 to 18 h after the end of surgery, hence, early postoperative changes may at that time be reversed. In agreement with other results cited by Ruel et al. we found that the ratio between resting blood flow and reactive hyperemia was similar in pre‐ and postoperative tests. These findings indicate that the mechanism for regulation of vascular resistance in the arm in response to acute physiological changes is intact after on‐pump cardiac surgery (Table 3). We suggest that reduced perfusion pressure with compensatory increased sympathetic vascular tone to maintain arterial pressure is the most likely mechanism for the reduction in brachial artery blood flow on the first postoperative morning.

In a pilot study, Sangalli et al. (2015) used FMD with flow measurements to examine the effect of CPB on endothelial responsiveness. The authors concluded that CPB with continuous flow impaired endothelial responsiveness while pulsatile flow CPB emerged as protective. Patients operated without CPB had an unchanged FMD. No significant change in brachial artery blood flow was found in either group from the preoperative test to the next morning. This study was, however, retrospective and small with only ten patients included in each study group. Preoperative FMD was within normal range, possibly indicating that this patient group is representative for patients with less advanced vascular disease. It is even more important that baseline measurements were made after induction of anesthesia; general anesthesia is known to affect myocardial function, cardiac output, sympathetic vascular tone, and peripheral vascular resistance; hence, baseline flow values acquired in general anesthesia may be incorrect.

## Limitations of the Study

There are limitations to our study: We included a cohort of low‐risk CABG patients and to reduce variation in the cohort patients with some common drugs and risk factors were excluded. Consequently, the applicability of our results is strictly speaking limited to this group of patients. However, it is reasonable to assume that the effect of CPB on peripheral blood flow and endothelial function is similar in other patient groups. A further potential limitation is that two recordings of excellent quality were required for each patient. This resulted in the exclusion of patients were we were unable to require satisfying image quality, mostly because the patient moved more than we were able to compensate for with the micrometer screw. However, patient movement is likely to appear at random and therefore the exclusion of these patients does not represent a systemic bias of results.

## Conclusion

The postoperative reduction in FMD found in this study was comparable to that observed in previous studies on this issue. Furthermore, this study reveals that reactive hyperemia, the stimulus to vessel dilation, was also reduced postoperatively. We conclude that the observed reduction in FMD may be a result of reduced shear stress on the vessel wall rather than a result of attenuated endothelial dysfunction.

Furthermore, the observed reduction in brachial artery blood flow is of clinical interest and justifies studies on postoperative blood flow distribution.

## Conflict of Interest

No competing interests.
